# The Role of Insulin Regulated Aminopeptidase in Endocytic Trafficking and Receptor Signaling in Immune Cells

**DOI:** 10.3389/fmolb.2020.583556

**Published:** 2020-10-20

**Authors:** Delphyne Descamps, Irini Evnouchidou, Vivien Caillens, Carole Drajac, Sabine Riffault, Peter van Endert, Loredana Saveanu

**Affiliations:** ^1^Université Paris-Saclay, INRAE, UVSQ, VIM, Jou-en-Josas, France; ^2^Université de Paris, Centre de recherche sur l’inflammation, INSERM U1149, CNRS ERL8252, Paris, France; ^3^Inovarion, Paris, France; ^4^Université de Paris, INSERM Unité 1151, CNRS UMR 8253, Paris, France; ^5^Service d’immunologie biologique, AP-HP, Hôpital Necker, Paris, France

**Keywords:** IRAP, endosome trafficking, immune cell responses, receptor signaling, formin, vacuolar protein sorting, TLR9, TCR

## Abstract

Insulin regulated aminopeptidase (IRAP) is a type II transmembrane protein with broad tissue distribution initially identified as a major component of Glut4 storage vesicles (GSV) in adipocytes. Despite its almost ubiquitous expression, IRAP had been extensively studied mainly in insulin responsive cells, such as adipocytes and muscle cells. In these cells, the enzyme displays a complex intracellular trafficking pattern regulated by insulin. Early studies using fusion proteins joining the IRAP cytosolic domain to various reporter proteins, such as GFP or the transferrin receptor (TfR), showed that the complex and regulated trafficking of the protein depends on its cytosolic domain. This domain contains several motifs involved in IRAP trafficking, as demonstrated by mutagenesis studies. Also, proteomic studies and yeast two-hybrid experiments showed that the IRAP cytosolic domain engages in multiple protein interactions with cytoskeleton components and vesicular trafficking adaptors. These findings led to the hypothesis that IRAP is not only a cargo of GSV but might be a part of the sorting machinery that controls GSV dynamics. Recent work in adipocytes, immune cells, and neurons confirmed this hypothesis and demonstrated that IRAP has a dual function. Its carboxy-terminal domain located inside endosomes is responsible for the aminopeptidase activity of the enzyme, while its amino-terminal domain located in the cytosol functions as an endosomal trafficking adaptor. In this review, we recapitulate the published protein interactions of IRAP and summarize the increasing body of evidence indicating that IRAP plays a role in intracellular trafficking of several proteins. We describe the impact of IRAP deletion or depletion on endocytic trafficking and the consequences on immune cell functions. These include the ability of dendritic cells to cross-present antigens and prime adaptive immune responses, as well as the control of innate and adaptive immune receptor signaling and modulation of inflammatory responses.

## IRAP Identification and Tissue Expression

The IRAP (insulin regulated or responsive aminopeptidase) protein is encoded by the human gene *LNPEP*. Although IRAP is the most commonly used name for this protein, other names related to the various substrates cleaved by the enzyme are also used, such as oxytocinase, OTASE, leucyl and cystinyl aminopeptidase, placental leucine aminopeptidase (P-LAP), cystinyl aminopeptidase (CAP), and vasopressinase.

While placental leucine aminopeptidase activities were described more than 50 years ago (Beckman et al., 1966, 1969), DNA sequences coding for IRAP were first identified in 1995 from a rat adipose tissue cDNA library (Keller et al., 1995) and 1 year later from a human placental cDNA library (Rogi et al., 1996). Soon after its identification, IRAP intracellular distribution was analyzed in adipocytes, where in basal conditions the enzyme colocalizes with the regulated glucose transporter, Glut4, in intracellular vesicles called Glut4 storage vesicles (GSV) (Ross et al., 1996). Upon adipocyte stimulation by insulin, IRAP, like Glut4, rapidly translocates to the cell surface from where it is rapidly endocytosed with a half-life of about 3 to 5 min, probably *via* a clathrin-mediated endocytic pathway (Summers et al., 1999). Even though the vast majority of studies on IRAP had been performed in adipocytes, already Keller et al. (1995) detected the protein by immunoblot in several tissues, such as heart, brain, spleen, lung, muscles, and kidney. The wide tissue distribution of IRAP has been confirmed by Mizutani’s group which detected IRAP at mRNA and protein level in placental syncytiotrophoblasts, endothelial cells, gastrointestinal tract, several epithelial cell types from the liver, pancreas, lung, and kidney, as well as neuronal cells (Nagasaka et al., 1997). They concluded that the broad tissue distribution of IRAP is suggestive for a much more complex and varied function of the protein beyond the regulation of oxytocin and vasopressin levels in the blood.

Further studies confirmed the almost ubiquitous IRAP expression and its involvement in a variety of physiological processes, such as antigen cross-presentation by major histocompatibility class I (MHC-I) molecules (Saveanu et al., 2009; Segura et al., 2009; Weimershaus et al., 2012), endosomal Toll-like receptor (TLR) signaling (Babdor et al., 2017), lactation (Tobin et al., 2014), cognition (Banegas et al., 2010; Elkins et al., 2017), and stress responses (Hernández et al., 2015). During the investigation of these various functions, it became evident that in all cell types analyzed, IRAP is localized in intracellular vesicles reminiscent of adipocyte GSV that we proposed to call “cell-specific storage endosomes” (Saveanu and van Endert, 2012).

## IRAP Protein Structure and Post-Translational Modifications

IRAP belongs to the M1 aminopeptidase family and has more than 40% sequence identity with the endoplasmic reticulum aminopeptidases ERAP1 (synonyms A-LAP, PILSAP) and ERAP2 (synonym L-RAP). Based on phylogenetic analyses, IRAP, ERAP1, and ERAP2 were classified in a distinct group of M1 aminopeptidases named “oxytocinase subfamily of M1 aminopeptidases” (Tsujimoto and Hattori, 2005). Even though all three enzymes are able to trim the N-terminus of antigenic peptides, they do so in different patterns consistent with the need to produce epitopes in different antigen presentation pathways: ERAP1 and ERAP2 mainly contributing to direct MHC-I presentation (Saveanu et al., 2005; Weimershaus et al., 2013) and IRAP to cross-presentation (Saveanu et al., 2009; Segura et al., 2009; Weimershaus et al., 2012). The elucidation of the crystal structures of IRAP, ERAP1, and ERAP2 in combination with biochemical studies revealed differences in substrate specificity and mechanism of action (Nguyen et al., 2011; Evnouchidou et al., 2012, 2014; Mpakali et al., 2015a,b, 2017a) that could also explain different antigen processing by different cell types (Segura et al., 2009; Weimershaus et al., 2012; Dinter et al., 2014; Mpakali et al., 2017b). IRAP was the last member of this subfamily to have its crystal structure resolved (Mpakali et al., 2015b). This delay was mainly due to the presence of a cytoplasmic and transmembrane domain, which make the expression and purification of the full-length protein very difficult, as well as its high degree of glycosylation, which is a major hurdle for the acquisition of high-quality crystals suitable for crystallography. Therefore, much information on the enzyme structure came earlier from biochemical studies.

IRAP is a type-II membrane-spanning protein that has a cytoplasmic domain composed of 109 amino acids, followed by a 23-amino acid transmembrane domain and an extracellular or intraluminal (depending on its localization inside the cell) 893-amino acid domain. The extracellular domain bears the Zn^2+^ binding and GAMEN motifs that are essential for the enzyme’s aminopeptidase activity (Keller et al., 1995). Unlike murine IRAP, the human protein bears a putative cleavage site for ADAM12 (F154/A155) that after proteolysis allows the release of a soluble form detectable in the serum during pregnancy (Iwase et al., 2001; Ofner and Hooper, 2002; Ito et al., 2004). The targeting motifs in the IRAP cytosolic tail responsible for intracellular distribution and trafficking were identified to be the di-Leucine motifs LL53,54 and LL76,77 (Keller et al., 1995). Contrary to a mutant for LL53,54 that was found to have no effect on trafficking, a mutant for LL76,77 had a very strong impact on trafficking, indicating this di-leucine motif as the one essential for this function. More specifically, this motif was essential for the initial entry of IRAP in the insulin-responsive compartment during biosynthesis but not for its recycling back to this compartment after endocytosis (Hou et al., 2006; Watson et al., 2008). Moreover, a study by Jordens et al. (2010) demonstrated that the IRAP cytosolic domain alone is able to reconstitute the normal intracellular distribution of Glut4 in adipocytes and that its role is specific for trafficking of storage endosomes, since the constitutive TfR^+^ recycling endosomes were not affected.

A later study where recombinant IRAP fragments were expressed in, and purified from, insect cells provided important information on IRAP structure (Ascher et al., 2011). Using size-exclusion chromatography and dynamic light scattering, the authors observed that the full-length soluble protein was completely dimerized, even after mutation of two key cysteine residues that could form disulfide bonds, suggesting an extended dimer interface. Dimerization could be important for the optimization of the enzyme’s aminopeptidase activity, similar to findings for heterodimers formed by its sister enzymes ERAP1 and ERAP2 (Evnouchidou et al., 2014). The study by Ascher et al. (2011) also proposed an interaction between the domain containing the catalytic site and the C-terminal domain, since a construct lacking the latter showed reduced activity indicating an activating or regulatory role for the C-terminal domain. Recombinant IRAP exhibited enhanced substrate affinity in the presence of DTT suggesting a potential better access to the active site upon disruption of internal disulfide bonds. Moreover, the authors found evidence for a second non-catalytic Zn^2+^ binding site whose role still remains to be investigated.

The first IRAP crystal structure was obtained by the group of Parker (Hermans et al., 2015) at a 3.02 Å resolution for soluble recombinant human IRAP expressed in insect cells. The IRAP structure has four domains. Domain I forms an extended β-sandwich and domain II adopts a thermolysin-like α/β fold. Domain III forms a β-sandwich fold and makes a bridge between domains II and IV, while domain IV is completely α-helical and shows extensive interactions with domain II. IRAP was found as a dimer both in solution and in the crystal, with a conformation intermediate between the open and closed state of ERAP1 (Kochan et al., 2011; Nguyen et al., 2011). Similar to aminopeptidases A and N, IRAP uses its C-terminal domain to dimerize. Since the hydroxyl group of Tyr549, which is crucial for the formation of the catalytic intermediate, was not in a position allowing it to form a hydrogen bond, the authors proposed that the obtained structure is a snapshot of an inactive state of the enzyme. They suggested that this structure corresponds to an enzyme-product complex, where the C-terminal domain swings out from the active site in order to allow product release. The S1 pocket could fit well all amino acids except for tryptophan, which explains the wider specificity spectrum of IRAP compared to ERAP1 or ERAP2. More importantly, the GAMEN loop adopted a completely different position compared to other M1 aminopeptidases, which explains the unique ability of IRAP to cleave cyclic peptides such as oxytocin and vasopressin. Another structure (3.3 Å) obtained by the group of Stratikos (Mpakali et al., 2015b) superposed completely with the first one. In this case, the authors used a mammalian expression system with permanently transfected glycosylation-deficient cells, since use of cells with normal glycosylation did not provide crystals of good quality. The group provided also a second structure with a substrate analog (3.4 Å) containing a phosphinic group in the place of the first peptide bond, which allowed the formation of crystals corresponding to a transition state analog. The peptide N-terminus was anchored at the catalytic site while IRAP remained in the same semi-closed state, suggesting a domain organization unaffected by ligand binding, contrary to what has been observed for ERAP1. Though there were no deep specificity pockets interacting with the substrate, most interacting residues belonged to domain II. The C-terminus of the peptide was found to interact with residues from domain IV (C-terminal domain), but molecular dynamics simulations showed that it has a high degree of plasticity, suggesting that there is no specific recognition of the C-terminus by IRAP, again contrary to ERAP1. The GAMEN loop showed no direct interactions with the substrate, therefore it seems not to be crucial for binding of linear peptides. Mapping of the A609T single nucleotide polymorphism (SNP) that has been associated with psoriasis and ankylosing spondylitis revealed an interaction with the hinge domain III of IRAP. Similar to the ERAP1 SNP K528R also associated with various autoimmune diseases, the IRAP SNP A609T reduced enzyme activity almost by half. The same group obtained more recently the first structure of IRAP with an inhibitor at 2.53 Å that displayed important differences compared to the previous ones (Mpakali et al., 2017b). This structure is closed, with domain IV juxtaposed against domains I and II, being very similar to the active closed ERAP1 structure and to the only structure available for ERAP2 (Evnouchidou et al., 2012). The internal cavity containing the catalytic site has no access to the solvent and a new specificity pocket is formed. The GAMEN loop adopts in this case a unique configuration depicting an active site with structural plasticity that could allow accommodation of a wide range of substrates, including cyclic peptides suited to the various biological functions of IRAP. The active site shows a capacity to bind a greater variety of antigenic precursors and its structural adaptability could explain why most ERAP1 inhibitors are also IRAP inhibitors but not vice versa.

IRAP can undergo many post-translational modifications including *N*-glycosylation, *S*-acylation, phosphorylation, ubiquitination, acetylation, and mono-methylation and of course the soluble form produced by proteolytic processing in pregnant women (Table 1). Apart from being highly glycosylated already at steady state, IRAP glycosylation is modulated under inflammatory conditions since it contains glycans regulated by TNFα, as shown in adipocytes treated with TNFα. These altered glycans may modulate the role of IRAP in GSV trafficking (Parker et al., 2016). IRAP could be phosphorylated *in vitro* by PKC-ζ both in insulin-stimulated rat adipocytes and in purified Glut4 vesicles. Phosphorylation occurred on two major sites, Ser80 and Ser91, with the former accounting for 80–90% of total phosphorylation, and was partially inhibited in the presence of a PKC-ζ pseudo-substrate. The fact that intracellular trafficking of certain recycling membrane proteins has been shown to be regulated by phosphorylation and that PKC-ζ inhibitors abolish Glut4 recruitment to the plasma membrane, suggests that IRAP phosphorylation could be important for GSV trafficking (Ryu et al., 2002). More recently, IRAP was also found to be phosphorylated on Ser91 after activation of human CD8^+^ T lymphocytes bearing a chimeric antigen receptor (Salter et al., 2018), which indicates a potential role of IRAP in the trafficking of these receptors used in immunotherapy.

**TABLE 1:** Post-translational modifications found in human IRAP and their known or potential effect on IRAP function.

Post-translational modification	Regulation of modification	Known or potential effect on function
*N*-Glycosylation (Wollscheid et al., 2009; Parker et al., 2016)	At steady state/inflammation: TNFα regulation	Adipocytes: modulation of GSV trafficking?
Phosphorylation (Ryu et al., 2002; Salter et al., 2018)	Ser80 and Ser91/PKC-ζ phosphatase	Adipocytes: important for GSV trafficking? CAR CD^8+^ T cells-role in CAR trafficking?
*S*-Acylation (Werno and Chamberlain, 2015; Evnouchidou et al., 2020)	Cys103 and Cys114	HEK 293T cells: normal localization T cells: interaction with TCR, unaltered intracellular localization
Soluble form (Ofner and Hooper, 2002)	Proteolytic processing	Human serum during pregnancy: oxytocin and vasopressin degradation
Mono-methylation (Larsen et al., 2016)	Arg904	Protein–protein interaction
Ubiquitination (Udeshi et al., 2013)	Several sites	Protein degradation
Acetylation (phosphosite.org)	Lys691	Regulation of chromatin structure, gene expression and protein function

IRAP was found to be *S*-acylated at 60% in 3T3-L1 adipocytes. The two *S*-acylated residues were identified to be Cys103, just upstream of the transmembrane domain, and Cys114 that lies in the cytoplasmic side of the transmembrane domain. This attachment of palmitate and other fatty acids to a cysteine could mediate membrane attachment of soluble proteins, regulate intracellular trafficking and also affect protein–protein interactions and protein stability. A triple IRAP mutant that cannot be *S*-acylated was found to have a normal localization in HEK 293T cells (Werno and Chamberlain, 2015). However, in a recent study (Evnouchidou et al., 2020), we found that in T cells IRAP S-acylation is crucial for its interaction with the T cell receptor (TCR), even though, similar to HEK 293T cells, *S*-acylation-deficient IRAP mutant showed an unaltered intracellular localization. Therefore, it would be interesting to study the effect of IRAP *S*-acylation in other cell types, taking into account the specific function of IRAP in each cell type.

## IRAP Protein Interactions

**FIGURE 1:**
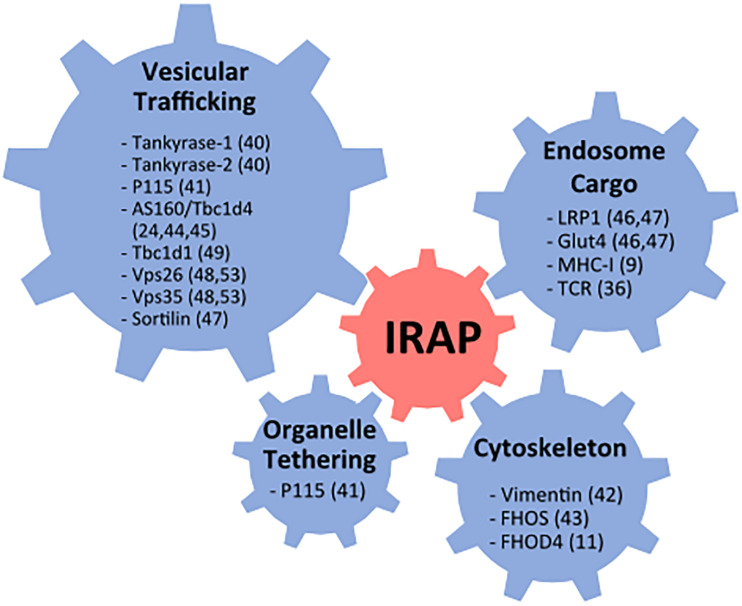
Summary of reported IRAP protein interactions. FHOS, formin homolog overexpressed in the spleen; Glut4, glucose transporter 4; MHC, major histocompatibility class; Vps, vacuolar protein sorting; TCR, T cell receptor.

Soon after its identification IRAP was found to interact with several proteins involved in vesicular trafficking, organelle tethering, and cytoskeleton remodeling (Figure 1). These include tankyrase-1, tankyrase-2 (Chi and Lodish, 2000), and p115 (Hosaka et al., 2005), three proteins involved in the regulation of Golgi vesicle trafficking, vimentin, an intermediate cytoskeleton filament (Hirata et al., 2011) and the actin remodeling protein FHOS [formin homolog overexpressed in the spleen (Tojo et al., 2003)]. In addition, IRAP was shown to interact with AS160/Tbc1d4, a Rab GTPase activating protein (GAP) specific for Rab8, 10, and 14, suggesting that IRAP participates in the recruitment of AS160 to endocytic membranes (Larance et al., 2005; Peck et al., 2006). However, further investigations did not confirm the role of the IRAP-AS160 interaction in AS160 recruitment to GSV in adipocytes (Jordens et al., 2010). In addition to these proteins involved in intracellular trafficking, IRAP was found to interact with proteins located in GSVs, such as sortilin, LRP1 and Glut4 in adipocytes (Shi et al., 2008; Kandror and Pilch, 2011), and with MHC-I in dendritic cells (DCs) (Saveanu et al., 2009). These protein interactions have been previously reviewed in Saveanu and van Endert (2012). The list of IRAP protein interactions has more recently been enriched by the discovery of its interaction with the ζ chain of the TCR as well as the Lck kinase in T lymphocytes (Evnouchidou et al., 2020) and by identification of other interactions relevant for IRAP’s role in vesicular trafficking, such as interaction with FHOD4, a formin similar to FHOS (Babdor et al., 2017), the retromer subunits Vps35 and Vps26 (Pan et al., 2019) and the Rab GAP Tbc1d1 (Mafakheri et al., 2018b).

FHOD4 is an actin-nucleation factor that assembles actin monomers into filaments (Kühn and Geyer, 2014) and might promote actin assembly on endosomes (Fernandez-Borja et al., 2005). We demonstrated that, in DCs, the interaction between FHOD4 and IRAP is required for anchoring the endosomes containing TLR9 and its ligands to the cell periphery and avoiding their fusion with lysosomes (Babdor et al., 2017).

The retromer is a large protein complex composed of a sorting nexin (SNX) dimer and a vacuolar protein sorting trimer (Vps26, Vps29, Vps35) that recycles diverse cargos from early endosomes to the *trans*-Golgi network or to the plasma membrane, preventing thus their transport to lysosomes (Chen et al., 2019). The SNX dimer is responsible for the retromer complex recruitment to the endosomal membrane, while the Vps trimer binds to various cargo molecules, among which IRAP has been recently identified (Pan et al., 2019). In the absence of the Vps35 subunit of the retromer, IRAP and Glut4 trafficking are perturbed and both proteins are found in lysosomes (Pan et al., 2017, 2019).

Tbc1d1 is a Rab GAP that is highly similar to Tbc1d4 and controls the activity of the same Rab proteins. When recruited to vesicular membranes, both Tbc1d4 and Tbc1d1 reduce the activity of Rab proteins involved in vesicle transport, thus mediating intracellular retention of IRAP vesicles. The interaction of Tbc1d1 with IRAP (Mafakheri et al., 2018a,b) suggests that IRAP participates in the mechanism of intracellular retention, not only in cells and tissues expressing Tbc1d4, but also in those expressing Tbc1d1. However, considering that in adipocytes depleted for IRAP, AS160 was still recruited to GSV (Jordens et al., 2010), the role of IRAP interactions with both Tbc1d4 and Tbc1d1 needs further investigation, not only in adipocytes, but also in other cell types.

## Regulation of IRAP Trafficking by Cell-Specific Cell Surface Receptors

In all the cells studied, IRAP is localized in intracellular vesicles, whose cargo varies depending on the cell type. In adipocytes, the main cargo is the regulated glucose transporter Glut4 and in neurons, the sst2A somatostatin receptor (De Bundel et al., 2015). In the immune system, where IRAP is expressed in almost all cells except neutrophils, IRAP vesicles contain MHC-I and TLR9 in DCs (Saveanu et al., 2009; Weimershaus et al., 2012; Babdor et al., 2017) or the ζ chain of the TCR in T cells (Evnouchidou et al., 2020). All these proteins display constitutive slow recycling and some of them also regulated trafficking, being transported to the cell surface under specific conditions. Thus, Glut4 translocates to the plasma membrane to increase glucose uptake under insulin stimulation (Antonescu et al., 2009) and the TCR ζ chain is rapidly transported to the plasma membrane under TCR activation by a cognate peptide/MHC-I complex (Evnouchidou et al., 2020).

**FIGURE 2:**
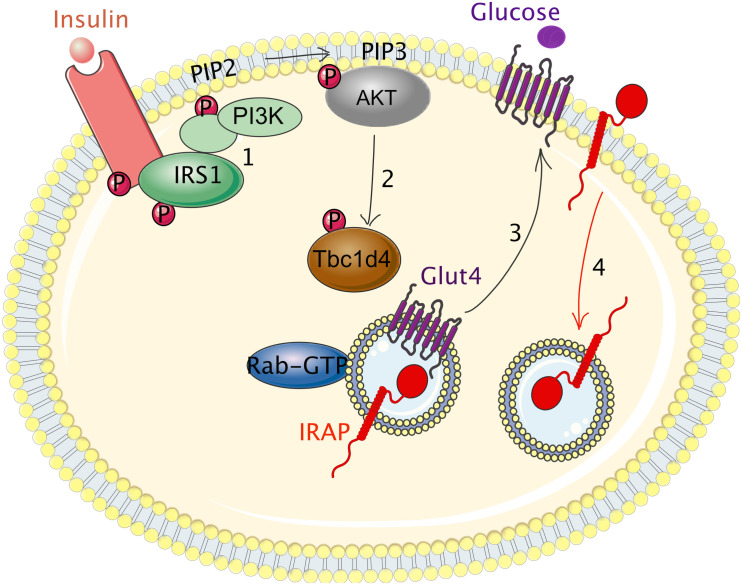
Regulation of IRAP trafficking by the insulin receptor in adipocytes. (1) Insulin binding to its receptor initiates a signaling cascade that induces the phosphorylation of insulin receptor substrate 1 (IRS1) on tyrosine residues (p-Tyr). p-Tyr in IRS1 serve as docking sites for class I-A PI3K. The interaction of PI3K with IRS1 activates PI3K leading to increased production of PIP3 at the inner leaflet of the plasma membrane that in turn leads to recruitment and activation of Akt. Akt phosphorylates Tbc1d4, the GAP for Rab14, Rab8, and Rab10 small GTPAses. (2) Tbc1d4 phosphorylation inactivates its GAP activity, allows Rabs activation and finally, induces GSV trafficking to the adipocyte cell surface (3). After Glut4 and IRAP translocation to the plasma membrane, Glut4 and IRAP rapidly re-internalize into intracellular vesicles (4). GTP, guanosine triphosphate; Glut4, glucose transporter 4; IRAP; insulin-regulated aminopeptidase; IRS1, insulin receptor substrate 1; GSV, Glut4 storage vesicles; PI3K, phosphoinositide 3-kinase; PIP2, phosphatidylinositol diphosphate; PIP3, phosphatidylinositol triphosphate; P, phosphorylation.

The molecular mechanisms driving cell surface translocation of IRAP vesicle cargos are by far best understood for adipocyte GSV (Figure 2). In basal conditions, GSV are retained intracellularly and their recycling to the plasma membrane is much slower than that of TfR (Zeigerer et al., 2004). Insulin binding to its receptor initiates a signaling cascade, in which class I-A PI3K plays a key role by triggering rapid translocation of GLUT4 and IRAP to the cell surface. By producing the PtdIns(3,4,5)P3 lipid at the inner leaflet of the plasma membrane, class I-A PI3K induces membrane recruitment of the kinases of the Akt/PKB family (Hopkins et al., 2020). Although all the members of the Akt family can be recruited to PtdIns(3,4,5)P3-rich membranes, Akt2 is the major kinase recruited to the plasma membrane upon insulin stimulation, as demonstrated by total internal reflection fluorescence (TIRF) microscopy and confirmed by Akt2 depletion or deletion (Gonzalez and McGraw, 2009a,b). Akt activity phosphorylates the GAPs AS160/Tbc1d4 and Tbc1d1, as discussed above (Mafakheri et al., 2018a). Phosphorylation inactivates the GAPs and allows activation of their Rab substrates. In adipocytes, Rab10 seems to be the main GTPase regulating Glut4 trafficking (Brewer et al., 2016), while in muscle cells this role seems to be played by Rab8 (Sun et al., 2014).

Although as yet there is no experimental evidence showing that the PI3K-AKT pathway and its downstream effectors, Tbc1d1/Tbc1d4, regulate IRAP vesicle trafficking in other cell types, recent data from our laboratory support the hypothesis that this may be the case in DCs (Weimershaus et al., 2018). In these cells, IRAP vesicle trafficking and translocation to the phagocytic cup is regulated by immune receptors, such as TLR4 and the receptors for the Fc fragment of immunoglobulins, FcγRs. Similar to insulin responsive tissues, the regulation of IRAP trafficking in DCs involves the GAP Tbc1d4 and its downstream effector Rab14, but the upstream kinases that phosphorylate Tbc1d4 in these cells remain to be identified.

By analogy with insulin responsive tissues and DCs, IRAP and the TCRζ chain trafficking might also be regulated by class I PI3K, which in T cells is activated downstream of the co-stimulatory receptor CD28 (Esensten et al., 2016). The downstream GAP and Rab effectors could differ between T lymphocytes and DCs, since the microarray data from the Immunological Genome Project^1^ predict that T cells express mainly Tbc1d1, Rab8, and Rab10, while DCs express foremost Tbc1d4 and Rab14 (Heng et al., 2008).

## IRAP Endosome Trafficking in Immune Cells

Immune cells display complex and highly regulated endocytic trafficking that is essential for triggering a correct immune response, avoiding inappropriate reactions that could lead to uncontrolled inflammation or autoimmune disease. Through its localization and protein interactions, IRAP participates in the regulation of diverse immunological processes described below.

### Accelerated Phagosomal Maturation in the Absence of Rab14

In immune cells, endocytosis or phagocytosis of pathogens is followed by maturation of internalized vesicles through fusion and fission with intracellular vesicles (Huotari and Helenius, 2011). Gradual changes in protein composition and pH result in degradation of the internalized material, while delayed or attenuated maturation promotes cross-presentation (Alloatti et al., 2015). Published mechanisms regulating maturation kinetics include incomplete assembly of the proton pump V-ATPase (Delamarre et al., 2005), recruitment of NADPH oxidase 2 (Savina et al., 2006) and LPS-induced Rab34-dependent perinuclear clustering of lysosomes (Alloatti et al., 2015). More recently, we proposed an additional mechanism regulating trafficking of early endosomes and phagosomes, with important consequences for phagosome maturation (Weimershaus et al., 2018). We found that, in bone marrow-derived DCs (BMDCs), IRAP colocalizes with Rab14 in peripheral endosomes and that both proteins are required for the formation and stabilization of GSV-like vesicles in these cells. Moreover, Rab14 knock-down led to accelerated phagosome maturation and enhanced killing of phagocytized *Pseudomonas aeruginosa*. Our data confirmed the previously published reports demonstrating that pathogens recruit Rab14 to slow down phagosome maturation through an unknown mechanism (Kyei et al., 2006; Kuijl et al., 2007; Capmany and Damiani, 2010; Hoffmann et al., 2014) and revealed the involvement of Rab14 in antigen cross-presentation.

In the absence of Rab14, cross-presentation of ova-anti-ova immune complexes by HEK293 cells transfected with FcγR and H2K^b^ was significantly decreased, while reconstitution with Rab14 in the same cells led to formation of enlarged GSV-like early endosomes and completely restored cross-presentation. Similar results were obtained in BMDCs, where Rab14 knock-down led to defective cross-presentation of both particulate and a soluble receptor-targeted ovalbumin antigen. Consistent with this, knock-out of Tbc1d4, the GAP for Rab14, resulted in formation of enlarged Rab14^+^Stx6^+^ endosomes and promoted cross-presentation. However, both a GTP-locked and a GDP-locked form of Rab14 reduced cross-presentation suggesting that the balance between active and inactive Rab14 and GTP hydrolysis itself are critical for cross-presentation.

**FIGURE 3:**
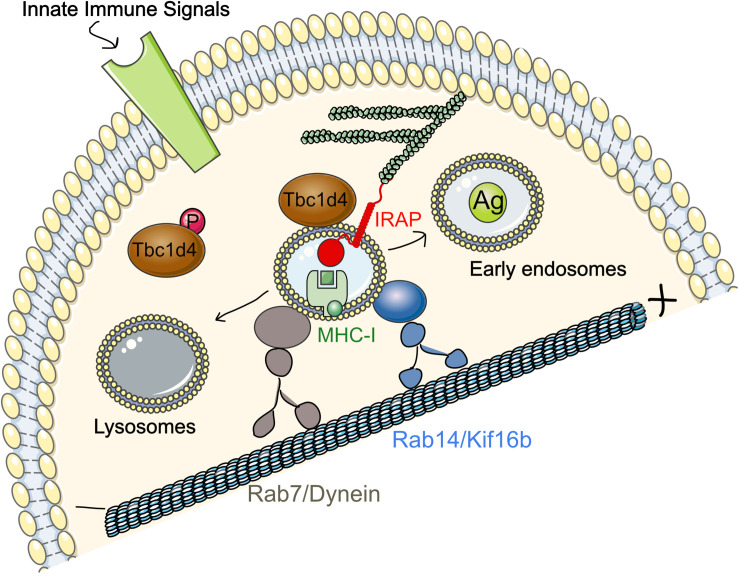
Regulation of IRAP and Rab14 trafficking by TLR4 and FcγR in DCs. In DCs, at steady state, IRAP colocalizes with Rab14 in peripheral endosomes and both proteins are required for the formation and stabilization of GSV-like vesicles in BMDCs. Signaling downstream of TLR4 or FcγR leads to Rab14 interaction with the kinesin KIF16b. Rab14/KIF16b protein complex promotes microtubule-guided anterograde transport of IRAP endosomes and their recruitment to phagosomes and early endosomes. Disruption of Rab14/KIF16b complex formation accelerates phagosome maturation and microbial killing, but compromises antigen cross-presentation activity of DCs. DCs, dendritic cells; FcγR, Fc gamma receptor; GSV, Glut4 storage vesicles; IRAP, insulin-regulated aminopeptidase; MHC-I, major histocompatibility class I.

We further demonstrated that the mechanism underlying the important role of Rab14 in cross-presentation is its interaction with the kinesin KIF16b (Weimershaus et al., 2018). Using a proximity ligation assay (PLA), we showed that this interaction is strongly enhanced upon immune complex binding to FcγR both in HEK293 expressing FcγR and in BMDCs after LPS stimulation. Our data suggest that IRAP is required for phagosomal recruitment of Rab14 (Figure 3). Thus, in the absence of IRAP, Rab14 failed to get recruited to early phagosomes and, in line with this result, maturation of IRAP-deficient phagosomes is not affected by Rab14 knock-down. The Rab14^+^ vesicles, as well as cross-presentation, could be restored in these cells through microtubule stabilization or dynein knock-down, while these cellular manipulations had little effect on wild type (WT) BMDCs. These results indicate that the dominant retrograde transport along microtubules in IRAP-deficient (IRAP KO) cells is responsible for the destabilization of Rab14^+^Stx6^+^ endosomes by favoring their fusion with lysosomes. In contrast, in WT cells, the anterograde motor formed by Rab14 and KIF16b ensures peripheral distribution of the endosomes, as demonstrated by the central distribution of Rab14 upon KIF16b knock-down. Moreover, KIF16b depletion led to reduced recruitment of Rab14 to phagosomes and defective cross-presentation, while it had no effect in IRAP KO BMDCs. In agreement with the results obtained by PLA, we showed that *in vitro* KIF16b interacts exclusively with GTP-locked Rab14.

Based on these results, we proposed a model in which, after receptor-mediated endocytosis or phagocytosis, Rab14^+^Stx6^+^IRAP^+^ vesicles are recruited to the internalized material (Figure 3). The signaling cascade engaged after FcγR or TLR4 activation leads to phosphorylation and therefore inactivation of Tbc1d4 that results in Rab14 activation. Rab14 can then bind GTP and allow formation of Rab14/KIF16b complexes that will promote anterograde transport of the vesicles. This will finally result in delayed antigen degradation and enhanced cross-presentation, two interconnected processes that both depend on IRAP and Rab14 (Weimershaus et al., 2018).

### IRAP Restricts TLR9-Driven Inflammatory Responses

Inappropriate activation of innate immune receptors, such as endosomal TLR7 or TLR9, promotes several autoimmune diseases, including psoriasis (Rahmani and Rezaei, 2016), a skin inflammatory disease that has been associated with single nucleotide mutations (A763T, A609T) in the *Lnpep* gene coding for IRAP. The A763T mutation generates a missense variant leading to decreased expression of the IRAP protein in skin lesions (Cheng et al., 2014). However, there is no direct evidence in humans that the IRAP protein regulates endosomal TLR activation in psoriasis. In contrast, in mice, we have demonstrated that IRAP controls the function of TLR9 (Babdor et al., 2017).

TLR9 is an innate immune receptor important for both innate and adaptive immune responses. Its activation directly induces innate immune responses, such as inflammatory cytokine and type I interferon (IFN) production. In parallel, by increasing the capacity of DCs to process and present antigens to lymphocytes, TLR9 indirectly participates in the generation of adaptive immune responses (Iwasaki and Medzhitov, 2004; Kawai and Akira, 2010). These responses are initiated by binding of pathogen-derived single-stranded DNA or self-derived nucleic acids to TLR9. While the recognition of pathogen DNA is crucial for the priming of immune responses, the recognition of self-DNA can generate autoimmune diseases. To prevent this, TLR9 activation is tightly regulated by intracellular receptor trafficking (Majer et al., 2017). At the steady state, TLR9 associated with the chaperon protein UNC93B1 is retained in the endoplasmic reticulum (ER) (Majer et al., 2019) whereas it translocates to endocytic vesicles after stimulation by TLR9 ligands, such as synthetic CpG-oligonucleotides. CpG dinucleotides are internalized *via* clathrin-mediated endocytosis in early endosomes, from where they are targeted to late endosomes containing the LAMP proteins (Latz et al., 2004). In addition, following cellular stimulation by TLR9 ligands, the TLR9-UNC93B1 complex is translocated to the cell surface, from where it is internalized in a clathrin-dependent manner through the interaction of UNC93B1 with the adaptor protein-2 (AP2) (Lee et al., 2013). The TLR9-UNC93B1 complex traffics to EEA1^+^VAMP3^+^ endosomes where TLR9 is processed by an array of proteases, such as cathepsins or the asparaginyl endopeptidase. This processing produces a carboxyterminal fragment able to bind the signaling adaptor Myd88 and to activate NF-κB-dependent proinflammatory cytokine production (Asagiri et al., 2008; Park et al., 2008; Sepulveda et al., 2009). Finally, the recruitment of AP-3 to the TLR9-UNC93B1 complex is mandatory for the trafficking of TLR9 to the lysosomal LAMP^+^ compartments where the IFN regulatory factor 7 (IRF7)-dependent signaling cascade is activated for type I IFN production (Sasai et al., 2010).

We recently found that IRAP interaction with the formin FHOD4 plays an important role in endosomal TLR9 trafficking and activation (Babdor et al., 2017). In the absence of IRAP and upon CpG activation of the cells, TLR9 signaling was amplified, leading to increased pro-inflammatory cytokine and type I IFN production in several DC subsets, such as BMDCs and splenic conventional and plasmacytoid DCs. IRAP-deficient mice displayed an exacerbated inflammatory response not only after CpG treatment, but also in the context of a model of respiratory infection (Babdor et al., 2017) with *P. aeruginosa*, a pathogen previously shown to activate TLR9 (Benmohamed et al., 2014). *P. aeruginosa* infection caused the death of IRAP-deficient animals, in experimental settings in which half of the WT animals survived. The death of IRAP-deficient animals was likely due to an uncontrolled inflammatory response since the broncho-alveolar lavage fluids from *P. aeruginosa*-infected IRAP-deficient mice contained higher concentrations of inflammatory cytokines (CXCL1, IL-6, TNF-α, and IL-1β) than that of WT mice. Moreover, upon *ex vivo P. aeruginosa* infection, alveolar macrophages isolated from IRAP-deficient mice secreted more IL-6 and TNF-α than their WT counterparts (Babdor et al., 2017).

By a combination of biochemical and cell biology methods, we demonstrated that TLR9 signaling is accelerated in IRAP-deficient BMDCs (Babdor et al., 2017). Enhanced TLR9 signaling was not due to IRAP’s enzymatic activity but was a consequence of aberrant TLR9 trafficking in the absence of IRAP. In WT cells, in basal conditions, TLR9 was localized in the ER and upon cell activation by CpG, TLR9 and CpG were internalized and retained for 2 h in IRAP^+^ endosomes. In contrast to WT cells, in IRAP-deficient cells, in basal conditions, TLR9 was found in LAMP^+^ lysosomes, in its processed form, ready to bind the signaling adaptors. In addition, upon cell activation, the TLR9 ligand was more rapidly targeted to lysosomes in IRAP-deficient cells compared to wild-type cells.

**FIGURE 4:**
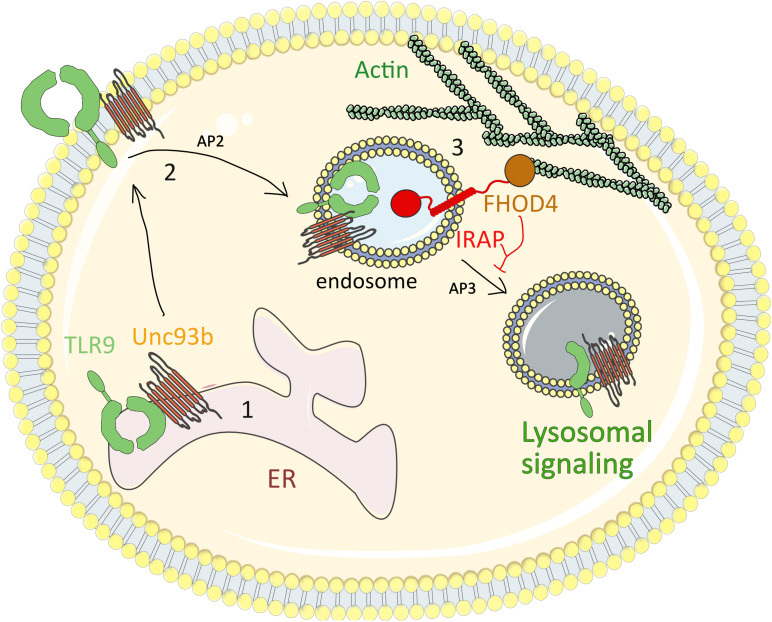
IRAP restricts TLR9-driven inflammatory response. (1) At steady state, TLR9 associated with the chaperon protein UNC93B1 is retained in the ER. (2) After cell stimulation by TLR9 ligands, such as synthetic CpG-oligonucleotides, the TLR9-UNC93B1 complex is translocated to the cell surface, from where it is internalized in a clathrin-dependent manner through the interaction of UNC93B1 with AP-2. (3) The internalized TLR9-UNC93B1 complex traffics to IRAP endosomes. IRAP interaction with the formin FHOD4 anchors the endosomes to the actin cytoskeleton and delays TLR9 trafficking to acidic endosomal compartments, where TLR9 undergoes a partial proteolysis and triggers the pro-inflammatory signaling cascades. AP2, adaptor protein-2; ER, endoplasmic reticulum; TLR9, Toll-like receptor 9; IRAP, insulin-regulated aminopeptidase.

To understand how IRAP retains TLR9 and its ligand away from lysosomes, we screened the previously published interactions of IRAP with cytoskeleton proteins and demonstrated that the cytosolic tail of IRAP interacts with the formin FHOD4, an actin-polymerization factor involved in anchoring vesicles to the actin cytoskeleton (Goode and Eck, 2007). Similar to IRAP deletion, FHOD4 depletion by RNA interference led to aberrant trafficking of TLR9 and increased production of pro-inflammatory cytokines upon cell stimulation by CpG. These data indicate that IRAP recruits FHOD4 to TLR9-containing endosomes. FHOD4 might drive actin polymerization around the endosomal compartments (Fernandez-Borja et al., 2005; Kühn and Geyer, 2014) which could delay the transport of TLR9-containing endosomes to lysosomes, thereby limiting TLR9 processing and activation. Thus, in DCs, IRAP controls TLR9 activation by delaying targeting of the receptor and its ligand to the acidic lysosomal compartments (Figure 4). Future experiments are required to establish if in addition to the IRAP/FHOD4 interaction, the recently discovered interaction between IRAP and the retromer (Pan et al., 2019) also participates in TLR9 retention away from lysosomes.

### Compromised TCR Signaling in the Absence of IRAP

The TCR complex is composed of the αβ, γε and δε heterodimers and a ζζ homodimer. The TCR initiates signaling cascades after recognition of a peptide-MHC (pMHC) complex, with low affinity recognition taking place in the thymus allowing for export to and survival in the periphery, and with high affinity interactions in initiation of effector T cell responses. The trafficking of TCR components and signaling partners is regulated in separate vesicular pools. While the α, β, γ, δ and ε chains are localized mainly in the ER, the nature of the ζ intracellular pool has been less well characterized (Soares et al., 2013; Alcover et al., 2018). Each of the γ, δ and ε chains bears one immunoreceptor tyrosine-based activation motif (ITAM), whereas each ζ chain contains three. In the absence of the ζ chain, TCR expression at the cell surface is almost abolished and there are substantial defects in T cell development (Love and Hayes, 2010; Alcover et al., 2018). Interestingly, signaling induced by the ζ chain is not necessary to reverse this situation but rather seems to be crucial for the activation of T cells in the periphery (Shores et al., 1994; Ardouin et al., 1999; Love and Hayes, 2010).

In a recent study (Evnouchidou et al., 2020), we investigated the nature of the intracellular ζ pool in Jurkat T cells and observed by immunofluorescence that it localizes in endosomes characterized by the presence of IRAP and Stx6. Moreover, IRAP could be co-immunoprecipitated with the ζ chain as well as with one of the first players in the TCR signaling pathway, the Lck kinase. We showed that at steady state, ζ chain endocytosis is controlled by the clathrin adaptor AP-2 and DnM2, while the absence of IRAP led to ζ chain accumulation at the cell surface, accompanied by higher expression of the whole TCR complex. To our surprise, despite higher TCR expression at the cell surface, IRAP-deficient Jurkat T cells presented a severe defect in TCR signaling as seen by diminished phosphorylation of various TCR signaling partners after activation. This result was in agreement with defective recruitment of these molecules to the immune synapse (IS) as seen by TIRF microscopy, and by confocal microscopy of conjugates of Jurkat T cells with Raji B cells loaded with the staphylococcal enterotoxin E (SEE) superantigen. Moreover, the conjugates with IRAP-deficient Jurkat T cells poorly stimulated IL-2 secretion, as measured by ELISA both in the case of the SEE superantigen and of a melanoma antigen.

**FIGURE 5:**
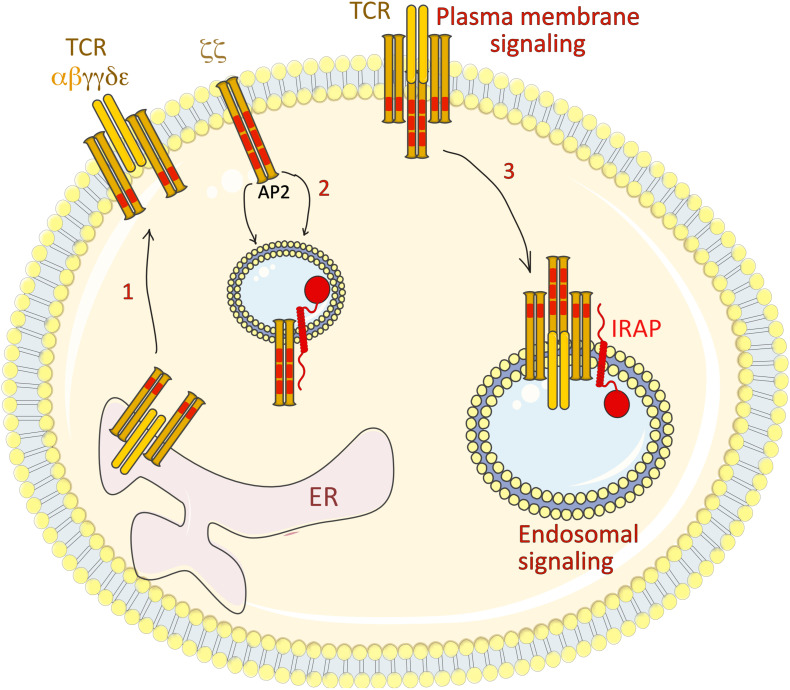
IRAP regulates TCR ζ chain trafficking and signaling. The full TCR comprises the clonotypic αβ chains, the γε and δε heterodimers and a ζζ homodimer. (1) While the αβ, γε and δε heterodimers assemble in the ER and are transported via the Golgi stacks to the plasma membrane, the majority of the ζζ homodimer is stored in IRAP intracellular vesicles. (2) In basal conditions, the ζ chain recycles between plasma membrane and the intracellular pool by a recycling pathway involving the clathrin adaptor AP2, the DnM2 and IRAP. Inhibition of this recycling pathway by AP2, DnM2 or IRAP depletion increases the levels of ζ chain at plasma membrane. (3) Under T cell activation, the ζζ homodimer is internalized in IRAP vesicles, from where it is able to trigger signaling cascades, as demonstrated by its interaction with several signalosome components, such as ZAP-70, Lck and LAT. AP2, adaptor protein-2; DnM2, dynamin-2; ER, endoplasmic reticulum; IRAP, insulin-regulated aminopeptidase; LAP, linker for activation of T cells; TCR, T cell receptor.

To investigate whether the observed defect could be attributed not only to a defective polarization of the intracellular pool to the IS but also to a reduced signaling capacity of the intracellular pool itself, we took advantage of a CD3ζ FRET- FLIM reporter molecule (Yudushkin and Vale, 2010). We observed a significant FRET reduction in IRAP-deficient Jurkat T cells, which was much more profound in the intracellular pool. Using the same reporter, we were able to visualize IS formation in live cells and observed a dynamic IS in WT cells being continuously supplied with CD3ζ from the intracellular pool, whereas the IRAP KO IS was rather static, getting CD3ζ mainly from the plasma membrane. In accordance with these results, Lck as well as pCD3ζ were also located at the plasma membrane in IRAP KO activated cells and IRAP vesicles were shown to contain various components of the TCR signalosome by PLA. We concluded that apart from TCR plasma membrane signaling, T cell activation necessitates the engagement of endosomal TCR signaling that takes place in IRAP + compartments (Figure 5).

Like in Jurkat T cells, IRAP colocalized with the TCR in mouse primary T cells and IRAP-deficient effector T cells presented increased TCR levels at the cell surface but defective signaling. Activation of the cells with a low affinity ligand resulted in similar initial cell division, but lower numbers of IRAP KO cells due to subsequent cell death. OT1 IRAP KO, as well as conditional IRAP KO in T cells, led to lymphopenia. In an effort to study whether this was due to a thymic selection defect, we checked IRAP expression and found that IRAP expression gradually increases through the stages of thymic selection and reaches its highest levels in the periphery. Moreover, there were no significant alterations observed in thymus subpopulations, which led us to the conclusion that the lymphopenic phenotype likely can be attributed to a reduced survival capacity in the periphery due to a defective tonic signal (Kirberg et al., 2001; Garbi et al., 2010). We finally showed that IRAP is important for the first wave of low affinity T cells in pathogen-specific T cell responses in the context of a tumor model where IRAP T cell conditional KO mice developed bigger tumors and were unable to initiate effective anti-tumor T cell responses. The results of this study could be used to ameliorate current T cell-based immunotherapies, as well as to investigate intracellular signaling of other immune receptors.

## Consequence of IRAP Deletion on Endocytic Trafficking in Other Cells

### IRAP as an Old Partner of Glut4 Transporter Trafficking

Soon after Glut4 discovery (Birnbaum, 1989; James et al., 1989), IRAP was identified as an abundant protein in biochemically isolated GSV (Keller et al., 1995). IRAP-deficient mice were produced for the first time in 2002 (Keller et al., 2002). These mice had normal levels of *Glut4* mRNA but showed reduced levels of Glut4 protein, with a reduction varying between 45% and 85% in different muscles and in adipocytes, indicating a role for IRAP in Glut4 expression. Interestingly, Glut4 deletion also reduced by 35% the amount of IRAP at protein level, but not its mRNA (Carvalho et al., 2004), indicating that both proteins participate in the regulation of GSV trafficking.

Further studies in differentiated 3T3-L1 adipocytes confirmed that IRAP knock-down disturbs Glut4 trafficking by increasing its levels at the plasma membrane in basal conditions, but does not affect Glut4 translocation upon insulin stimulation of the cells (Jordens et al., 2010). However, Glut4 trafficking seems to be slightly different between differentiated 3T3-L1 adipocytes and primary adipocytes, in which IRAP deletion does not increase the cell surface levels of Glut4, but reduces its protein amount (Keller et al., 2002). Differences in Glut4 trafficking between differentiated 3T3-L1 and primary adipocytes are also suggested by a more recent report in which IRAP-depleted differentiated 3T3-L1 adipocytes did not show a strong reduction of Glut4 protein (Pan et al., 2019). However, this study detected a significant shift of Glut4 from the endosomal pool to acidic lysosomal compartments, similar to that observed for TLR9 in IRAP KO DCs (Babdor et al., 2017). The lysosomal localization of Glut4 in the absence of IRAP might be explained by the interaction between IRAP and the retromer (Pan et al., 2019). By interacting with the retromer, IRAP can cooperate with sortilin, another component of GSV that interacts with the retromer (Pan et al., 2017), and rescue Glut4 from degradation in lysosomes. This phenomenon could be particularly relevant in primary cells, where IRAP deletion strongly reduced Glut4 protein levels (Keller et al., 2002), probably due to its degradation in lysosomes.

### Somatostatin Receptors

In neurons, the major somatostatin receptor subtype, sst2A, has been found in IRAP vesicles (De Bundel et al., 2015). Sst2A is an inhibitory receptor that reduces neuron excitability and has anticonvulsant effects (Schonbrunn, 1999). Like the majority of G-protein-coupled receptors, sst2A is rapidly internalized after ligand binding and undergoes slow recycling through the *trans*-Golgi network, avoiding lysosomal degradation. When rat hippocampal neurons were incubated with the sst2A agonist octreotide, the sst2A receptor was rapidly internalized in IRAP endosomes from where it was recycled back to the plasma membrane and reached the initial expression levels at the plasma membrane between 1 and 2 h after cell stimulation. IRAP depletion by lentiviral shRNA accelerated the recycling of the receptor that recovered its normal expression at the plasma membrane within 45 min. Interestingly, angiotensin IV and the LVV-H7 compound, two previously known IRAP ligands, also increased the speed of sst2A receptor recycling, similar to IRAP depletion. Further experiments are required to investigate if angiotensin IV or LVV-H7 binding to IRAP disturb IRAP intracellular trafficking and by consequence, sst2A receptor recycling.

## Conclusion and Perspectives

A growing number of experimental data, partially reviewed in this article, show that next to its aminopeptidase activity, IRAP is a regulator of endosomal trafficking. IRAP protein interactions with cytoskeleton components and with the retromer contribute to the regulation of cell specific receptor trafficking and activation. The majority of these receptors are involved in cellular adaptation to the environmental conditions, such as high glucose concentration for Glut4, bacterial infections for TLR9, increased neuronal excitability for sstA2 or anti-tumor activity of T lymphocytes for the TCR. Identification of other cell-specific cargos of IRAP endosomes in the future will complete the list of physiological and pathological conditions in which IRAP might help the organism to maintain its homeostasis. Whether the enzymatic activity of IRAP, next to its undisputed and independent role in processing substrates such as vasopressin, also matters in its role in vesicle trafficking remains to be explored further. Enzymatic activity is not involved in the regulation of TLR9 or TCR trafficking and function, and Glut4 trafficking in IRAP KO cells is restored by the expression of the cytosolic domain of IRAP that lacks aminopeptidase activity (Jordens et al., 2010). However, the example of modulation of sstA2 trafficking by IRAP inhibitors (De Bundel et al., 2015) suggests that IRAP inhibitors might change protein trafficking, a hypothesis that is worth future studies.

Further studies are also required to extend our knowledge on the cell surface receptors and the downstream signaling pathways that regulate IRAP vesicles trafficking in the immune system. For example, our recent results demonstrate that IRAP trafficking is affected by TLR4 and FcRs activation in DCs (Weimershaus et al., 2018), and by TCR activation in T cells (Evnouchidou et al., 2020). Class I PI3K are known to be activated by the signaling cascades downstream of TLR4 and FcRs (Ben Mkaddem et al., 2019; Fitzgerald and Kagan, 2020), as well as through the signaling downstream of T cell costimulatory receptors and the IL-2 receptor (Saravia et al., 2020). Therefore, PI3K/AKT signaling could be a regulator of IRAP trafficking in immune cells, similar to its role in adipocytes (Li et al., 2019). This hypothesis is compatible with the results available for immune cells and adipocytes but needs further experimental validation.

## Author Contributions

DD, IE, VC, CD, SR, PE, and LS wrote the manuscript. All authors contributed to the article and approved the submitted version.

## Conflict of Interest

The authors declare that the research was conducted in the absence of any commercial or financial relationships that could be construed as a potential conflict of interest.

## Footnotes

1 http://www.immgen.org/
